# Two Cases of Sinonasal Non-Intestinal-Type Adenocarcinoma with Squamoid Morules Expressing Nuclear *β*-Catenin and CDX2: A Curious Morphologic Finding Supported by Molecular Analysis

**DOI:** 10.1155/2018/8741017

**Published:** 2018-09-13

**Authors:** Tatiana M. Villatoro, Stacey K. Mardekian

**Affiliations:** Department of Pathology, Anatomy and Cell Biology, Thomas Jefferson University Hospital, Philadelphia, PA 19107, USA

## Abstract

Sinonasal non-intestinal-type adenocarcinoma (non-ITAC) is a rare, morphologically diverse neoplasm of the head and neck. Squamoid morular metaplasia has recently been reported as an occasional finding in non-ITAC. Interestingly, these squamoid morules often show aberrant expression of CDX2 as well as nuclear expression of *β*-catenin, similar to other tumors that show this type of metaplasia, but the underlying mechanism responsible for this finding is not completely understood. We present two cases of low-grade non-ITAC with squamoid morules coexpressing CDX2 and nuclear *β*-catenin by immunohistochemistry, both of which were found to harbor a mutation in* CTNNB1*, the gene encoding *β*-catenin. This finding provides support that an alteration in the *β*-catenin pathway, including mutations in the *β*-catenin gene itself, is responsible for this recently described morphologic phenomenon in non-ITAC.

## 1. Introduction

The sinonasal tract is affected by a diverse spectrum of neoplasms, which may be epithelial, mesenchymal, or neuroectodermal in origin [1]. Adenocarcinomas in this site include salivary type and non-salivary type, which is further subclassified as intestinal type and non-intestinal type. Sinonasal intestinal-type adenocarcinoma (ITAC) is morphologically similar to adenocarcinomas of intestinal origin and is characterized by the immunohistochemical expression of intestinal markers, such as cytokeratin (CK) 20, CDX2, and villin [2]. Sinonasal non-intestinal-type adenocarcinoma (non-ITAC) is a rare type of adenocarcinoma that lacks a salivary or intestinal morphology, and thus is typically regarded as a diagnosis of exclusion, although recent studies have confirmed seromucinous differentiation in the majority of these tumors [3]. Non-ITAC is morphologically diverse, with architectural patterns ranging from papillary, tubular, solid or nested [1]. Furthermore, squamoid morular metaplasia has been recently described as an occasional finding in non-ITAC [3]. These squamoid morules have been shown to express aberrant CDX2 by immunohistochemistry (IHC), which may potentially pose a diagnostic pitfall. Additionally, nuclear expression of *β*-catenin in the squamoid morules has been reported in non-ITAC and other tumors showing this type of metaplasia [3]. Although this finding suggests some alteration in the Wnt/*β*-catenin signaling pathway, molecular characterization of non-ITAC is limited. We present two cases of non-ITAC with squamoid morules coexpressing CDX2 and nuclear *β*-catenin, which were found to have* CTNNB1* mutations detected by next-generation sequencing (NGS).

## 2. Case Presentation

### 2.1. Case 1

**Figure 1:**
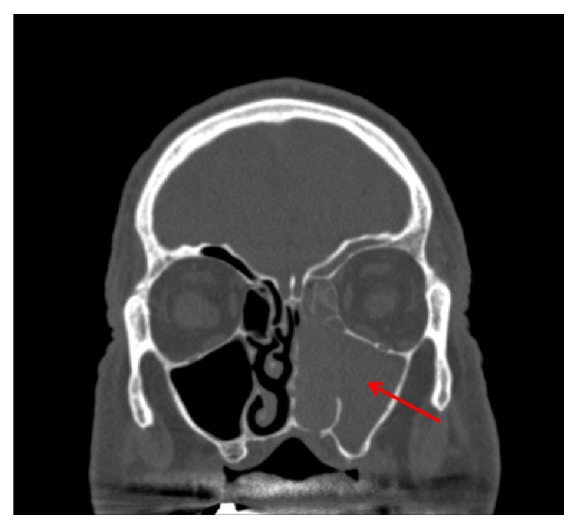
CT sinuses (coronal view) for Case 1. There is contiguous opacification of the left maxillary sinus, nasal cavity, ethmoid air cells, and frontal sinus.

An 80-year-old man with a past medical history of diabetes mellitus presented to his primary care physician after several weeks of left-sided congestion and epistaxis that he had attributed to seasonal allergies. He denied significant weight loss, headaches, visual changes or weakness. A computed tomography (CT) scan of the sinuses showed abnormal soft tissue causing contiguous opacification of the left nasal cavity, frontal, maxillary and ethmoid sinuses (Figure 1).

After a biopsy confirmed carcinoma, the patient was referred for further surgical management. A repeat CT confirmed a 1.5 cm polypoid opacity in the superior left nasal cavity with likely involvement of the anterior cranial base. The patient then underwent an endoscopic craniofacial resection of the sinonasal mass.

**Figure 2:**
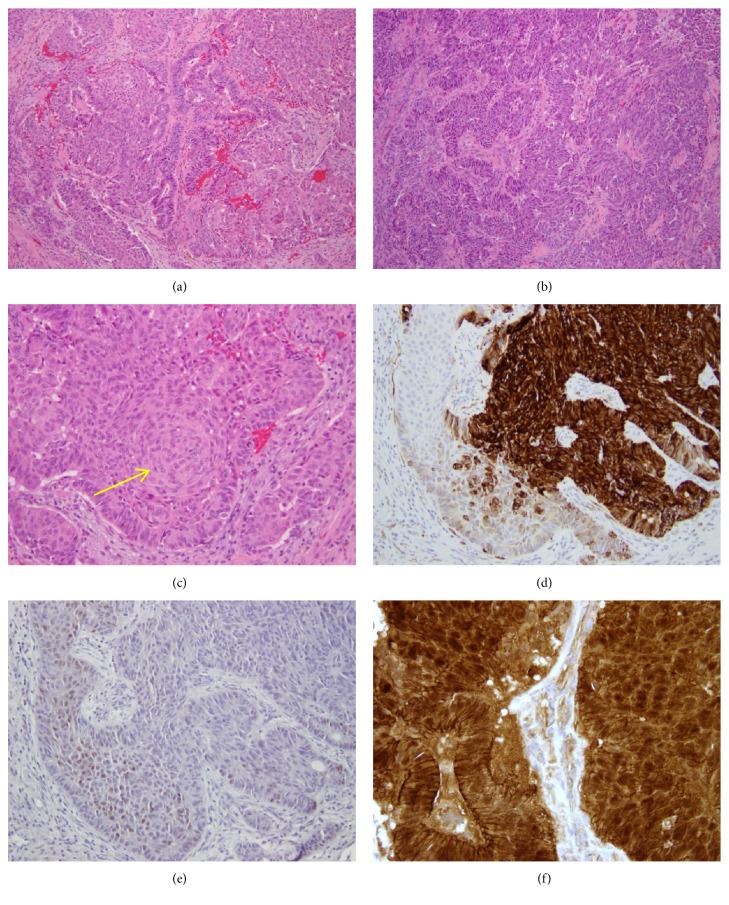
Morphologic and immunohistochemical findings in Case 1. The tumor is composed of a mixture of back-to-back glands, anastomosing cords, and solid areas with squamoid morular metaplasia ((a) and (b), H&E, x100). Squamoid morular metaplasia is easily found ((c), H&E, x200). CK7 is positive in areas of glandular differentiation and negative in squamoid morules ((d), x200). CDX2 is positive in squamoid morules and negative in glandular areas ((e), x200). *β*-catenin shows diffuse membranous staining throughout the tumor and nuclear staining restricted to squamoid morules ((f), x400).

Grossly, the specimen was received as multiple fragments of pink-purple ragged soft tissue measuring 3.5 cm in aggregate. Microscopic examination revealed a low-grade sinonasal non-ITAC consisting of a mixture of back-to-back glands, anastomosing cords, and solid areas with squamoid morular metaplasia (Figures 2(a)–2(c), x100). Immunohistochemical stains showed that the tumor cells were positive for CK7 in the areas of glandular morphology (Figure 2(d)), and they were negative for CK20, SOX10, CK5/6, p40, p63, and DOG1. CDX2 showed focal weak nuclear staining exclusively within the squamoid morules (Figure 2(e)), and *β*-catenin likewise showed nuclear staining restricted to squamoid morules with membranous staining throughout the rest of the tumor (Figure 2(f)).

Next-generation sequencing was performed by Foundation Medicine (Cambridge, MA). The analysis identified a missense mutation (S37C) in* CTNNB1*, the gene encoding *β*-catenin.

The patient received postoperative radiotherapy and has no evidence of disease at 10 months after surgery.

### 2.2. Case 2

**Figure 3:**
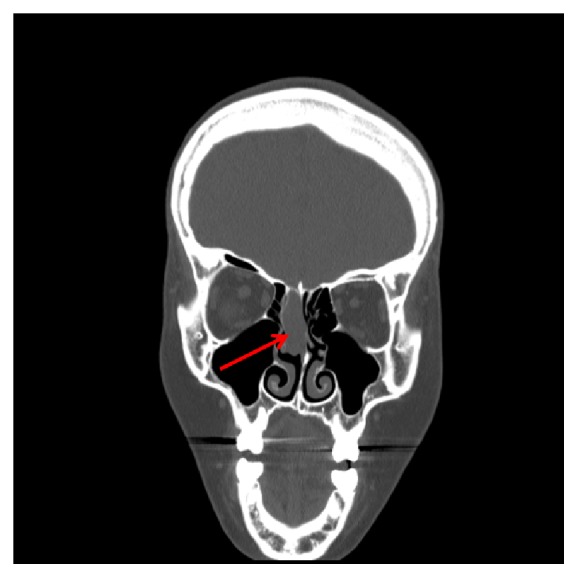
CT sinuses (coronal) for Case 2. There is a 3 cm ovoid opacity in the mid to superior aspect of the right nasal cavity with extension to the roof of the nasal cavity.

A 25-year-old Asian female with a past medical history of myotonic muscular dystrophy presented to an otolaryngologist with complaints of otalgia. She reported intermittent right ear pain of mild severity, as well as sneezing, itchy nose, and watery eyes for one month. Nasal endoscopy revealed a lobulated, fleshy mass just medial to the right middle turbinate. A CT scan of the sinuses revealed a 3.0 cm mass in the right nasal cavity extending to the roof of the nasal cavity, without an obvious cranial base defect (Figure 3).

**Figure 4:**
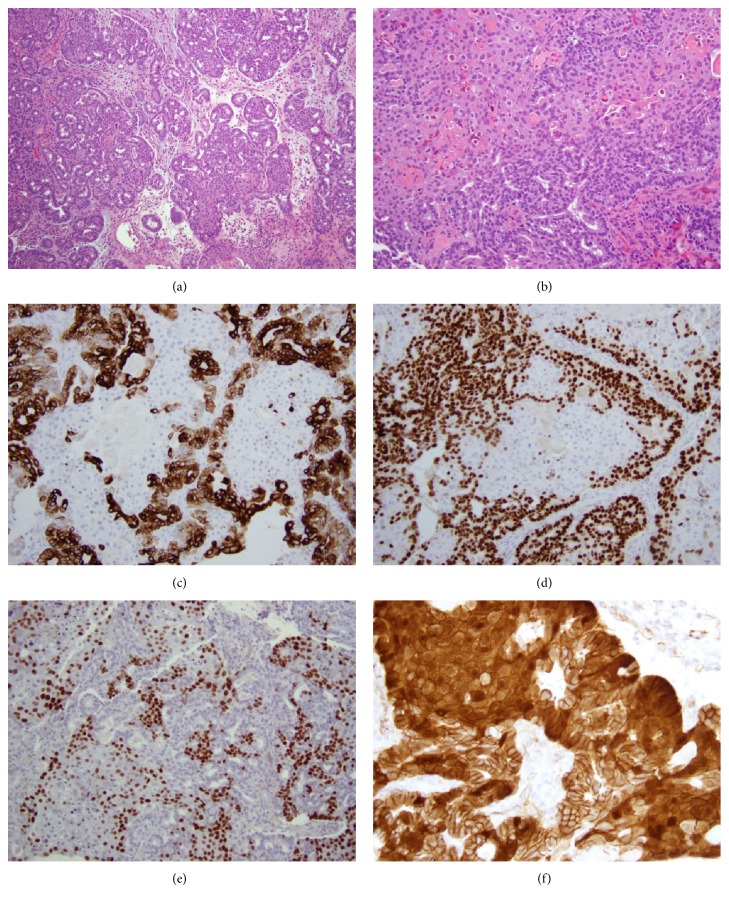
Morphologic and immunohistochemical findings in Case 2. The tumor is composed of a tubulolobular proliferation of cuboidal cells with minimal cytologic atypia, and interspersed areas of squamoid morular metaplasia ((a), H&E, x100). Other areas of the tumor show more confluent squamoid metaplasia ((b), H&E, x200). CK7 positivity is restricted to glandular areas of the tumor ((c), x200). SOX10 is likewise positive in glandular areas and negative in squamoid areas ((d), x200). CDX2 shows an inverse staining pattern to CK7 and SOX10, with positivity restricted to squamoid areas ((e), x200). Nuclear accumulation of *β* catenin is seen in squamoid areas ((f), x400).

It was unclear by imaging characteristics whether the mass represented a nasal polyp, papilloma, or malignant tumor. A biopsy was then performed, which revealed a proliferation of cytologically bland cuboidal cells forming crowded glandular structures, together with many squamoid morules as well as more confluent areas of squamoid metaplasia (Figures 4(a) and 4(b)). Immunohistochemical stains showed tumor cell expression of CK7 and SOX-10 in the glandular but not squamoid areas of the tumor (Figures 4(c) and 4(d)). CK20, CK5/6, and DOG1 were negative. Nuclear *β*-catenin and CDX2 were strongly expressed in the areas of squamoid morular metaplasia (Figures 4(e) and 4(f)).

These findings supported the diagnosis of a low-grade sinonasal non-ITAC. NGS was performed by Foundation Medicine, which detected a missense mutation (S33C) in* CTNNB1*.

The patient subsequently underwent endonasal craniofacial resection of the tumor to negative margins. Given the low-grade histology and the pathologic stage of T1N0M0, the patient did not require adjuvant chemotherapy or radiation and will undergo close clinical surveillance.

## 3. Discussion

Sinonasal non-ITAC is a rare yet phenotypically diverse neoplasm of the head and neck which occurs over a wide age range [1]. Low-grade non-ITAC is usually papillary and/or tubular in architecture and composed of cuboidal to columnar epithelial cells [1]. High-grade tumors may show solid growth with occasional glandular structures, or a nested infiltrative pattern with increased mitotic activity and necrosis [1]. Some cases of non-ITAC may consist primarily of clear cells arranged in nests, resembling clear cell carcinoma of the kidney, and have therefore been referred to as sinonasal renal cell-like carcinoma [1]. In addition, a recent series [3] that compared clinicopathologic and immunophenotypic features of ITAC, non-ITAC, and sinonasal seromucinous hamartomas (SSH) noted the peculiar presence of squamoid morular metaplasia in several cases of non-ITAC (as well as in SSH), while none of the ITAC cases showed this finding. Specifically, 4 out of 22 (17%) cases of non-ITAC showed squamoid morular metaplasia. Interestingly, 3 of 20 (15%) cases also showed expression of CDX2 limited to the squamoid morules. CDX2 is a homeobox gene that encodes a nuclear transcription factor involved in intestinal differentiation [4]. The expression of CDX2 introduces a potential diagnostic pitfall since ITAC most often demonstrates an intestinal immunophenotype (negative for CK7, positive for CDX2, CK20, and villin), whereas non-ITACs are consistently positive for CK7 and expected to be negative for CK20 and CDX2 [3]. The distinction between ITAC and non-ITAC is critical, as the two neoplasms have different clinical behavior and prognosis [5].

Diffuse and weak positivity for CDX2 has previously been noted in several other types of sinonasal neoplasms. Tilson et al. [5] found that 6 of 16 (38%) cases of sinonasal undifferentiated carcinoma, 8 of 81 (10%) cases of squamous cell carcinoma (both keratinizing and non-keratinizing), 2 of 20 (10%) cases of salivary-type adenocarcinoma cases, and 1 of 2 (50%) cases of small cell carcinoma showed some degree of CDX2 immunoexpression [5]. This further demonstrates that CDX2 is not by itself a reliable indicator of intestinal differentiation and should always be combined with CK20 in the immunohistochemical work-up of sinonasal malignancies.

Purgina et al. [3] also noted that the squamoid morules in 3 non-ITAC cases showed coexpression of nuclear *β*-catenin and CDX2. *β*-catenin is part of the cadherin protein complex, which plays a role in cell-cell adhesion, and it is normally degraded through the ubiquitin-proteosome mechanism [6]. When the *β*-catenin gene* CTNNB1 *is mutated or another process results in upregulation of the Wnt pathway, the degradation of *β*-catenin is reduced and it accumulates in the cytoplasm and nucleus and acts a transcription factor [6]. Interestingly, aberrant expression of CDX2 and *β*-catenin in morular metaplasia has also been noted in other neoplasms of various organs, including endometrioid ovarian carcinoma [6], endometrial carcinoma [7], and pulmonary blastoma [8]. This suggests that both CDX2 and *β*-catenin may play a role in squamoid morule formation at the molecular level.

Previous studies have detected* CTNNB1* mutations in tumors demonstrating squamoid morules strongly positive for nuclear and cytoplasmic *β*-catenin immunohistochemical expression [9]. Sekine et al. [8] reported two cases of pulmonary blastoma and three cases of well differentiated fetal adenocarcinoma of the lung with squamoid morules which had somatic missense mutations in the *β*-catenin gene. As described by Sekine et al., these mutations are “considered to result in *β*-catenin stabilization and constitutive activation of Tcf/Lef-dependent transcription.” Additionally, Saegusa and Okayasu [10] reported missense mutations in exon 3 of the *β*-catenin gene, involving codons 32, 33, 34, 37, 41, and 45, in 22 of 26 (84.6%) cases of endometrioid endometrial cancer with squamous morules. However, a genetic alteration in the *β*-catenin pathway to explain the morular formation in sinonasal non-ITAC, to our knowledge, has yet to be reported.

Molecular analysis has revealed defining mutations in other tumors occurring in the sinonasal tract. Similar gain-of-function mutations in* CTNNB1* are characteristic of sinonasal glomangiopericytoma, a distinctive mesenchymal neoplasm with a myoid phenotype [11]. Additionally, a recent study described three cases of low-grade non-ITAC showing ETV6 gene rearrangements, including two cases with ETV6-NTRK3 fusion and one with ETV6-RET fusion [12, 13]. While these gene fusions are also characteristic of secretory carcinoma, ETV6-rearranged low-grade sinonasal adenocarcinoma is a morphologically distinct entity [13, 14]. With continued investigation, other tumors falling into the category of non-ITAC may be further separated into more specific entities. Yom et al. [15] noted a small subset of non-ITAC cases showing p53 overexpression, whereas other cases did not show any genetic abnormalities in* KRAS*,* APC*,* CTNNB1*, DNA mismatch repair genes, or* TP53*. Another study by Franchi et al. [16] reported a subset of non-ITAC cases with EGFR overexpression, while two cases contained a BRAF mutation by direct sequencing. Now we report two cases of non-ITAC with mutated* CTNNB1* and a unique morphology and immunophenotype.

Clearly, non-ITAC is diverse not only morphologically but at the molecular level. With an advanced understanding in the molecular basis of non-ITAC comes the potential improvement in treatment options for patients. Several clinical trials are currently underway and preclinical evidence suggests that tumors with activating* CTNNB1 *mutations may be responsive to mTOR inhibitors [17] and inhibition of the NOTCH signaling pathway by gamma-secretase inhibitors [18]. With continued molecular investigation, the tumors encompassed by this diagnostic category of exclusion will be further subclassified, and this will hopefully lead to refinement of treatment strategies.

## 4. Conclusion

Squamoid morules are an interesting morphologic feature and can be present in non-ITAC, and they show a peculiar coexpression of CDX2 and *β*-catenin that suggests a possible synergistic effect in squamoid morular formation. We report two cases with a confirmed mutation in *β*-catenin which can provide a molecular explanation for this recently described morphological phenomenon in non-ITAC.

## References

[1] El-Naggar A. K., Chan J. K. C., Grandis J. R., Takata T., Slootweg P. J. (2017). *WHO Classification of Head and Neck Tumours*.

[2] Kennedy M. T., Jordan R. C. K., Berean K. W., Perez-Ordoñez B. (2004). Expression pattern of CK7, CK20, CDX-2, and villin in intestinal-type sinonasal adenocarcinoma. *Journal of Clinical Pathology*.

[3] Purgina B., Bastaki J. M., Duvvuri U., Seethala R. R. (2015). A subset of sinonasal non-intestinal type adenocarcinomas are truly seromucinous adenocarcinomas: a morphologic and immunophenotypic assessment and description of a novel pitfall. *Head & Neck Pathology*.

[4] Li M. K., Folpe A. L. (2004). CDX-2, a new marker for adenocarcinoma of gastrointestinal origin. *Advances in Anatomic Pathology*.

[5] Tilson M. P., Gallia G. L., Bishop J. A. (2014). Among sinonasal tumors, CDX-2 immunoexpression is not restricted to intestinal-type adenocarcinomas. *Head & Neck Pathology*.

[6] Houghton O., Connolly L. E., McCluggage W. G. (2008). Morules in endometrioid proliferations of the uterus and ovary consistently express the intestinal transcription factor CDX2. *Histopathology*.

[7] Wani Y., Notohara K., Saegusa M., Tsukayama C. (2008). Aberrant Cdx2 expression in endometrial lesions with squamous differentiation: important role of Cdx2 in squamous morula formation. *Human Pathology*.

[8] Sekine S., Shibata T., Matsuno Y. (2003). *β*-catenin mutations in pulmonary blastomas: association with morule formation. *The Journal of Pathology*.

[9] Makishi S., Kinjo T., Sawada S. (2006). Morules and morule-like features associated with carcinomas in various organs: report with immunohistochemical and molecular studies. *Journal of Clinical Pathology*.

[10] Saegusa M., Okayasu I. (2001). Frequent nuclear *β*-catenin accumulation and associated mutations in endometrioid-type endometrial and ovarian carcinomas with squamous differentiation. *The Journal of Pathology*.

[11] Lasota J., Felisiak-Golabek A., Aly F. Z., Wang Z.-F., Thompson L. D. R., Miettinen M. (2015). Nuclear expression and gain-of-function *β*-catenin mutation in glomangiopericytoma (sinonasal-type hemangiopericytoma): insight into pathogenesis and a diagnostic marker. *Modern Pathology*.

[12] Andreasen S., Skálová A., Agaimy A. (2017). ETV6 gene rearrangements characterize a morphologically distinct subset of sinonasal low-grade non–intestinal-type adenocarcinoma. *The American Journal of Surgical Pathology*.

[13] Andreasen S., Kiss K., Melchior L. C., Laco J. (2018). The ETV6-RET gene fusion is found in ETV6-rearranged Low-grade sinonasal adenocarcinoma without NTRK3 involvement. *The American Journal of Surgical Pathology*.

[14] Skalova A., Vanecek T., Martinek P. (2018). Molecular profiling of mammary analog secretory carcinoma revealed a subset of tumors harboring a novel ETV6-RET translocation: report of 10 cases. *The American Journal of Surgical Pathology*.

[15] Yom S. S., Rashid A., Rosenthal D. I. (2005). Genetic analysis of sinonasal adenocarcinoma phenotypes: distinct alterations of histogenetic significance. *Modern Pathology*.

[16] Franchi A., Innocenti D. R. D., Palomba A. (2014). Low prevalence of K-RAS, EGF-R and BRAF mutations in sinonasal adenocarcinomas. Implications for anti-EGFR treatments. *Pathology & Oncology Research*.

[17] Fujishita T., Aoki K., Lane H. A., Aoki M., Taketo M. M. (2008). Inhibition of the mTORC1 pathway suppresses intestinal polyp formation and reduces mortality in ApcΔ716 mice. *Proceedings of the National Acadamy of Sciences of the United States of America*.

[18] Shang H., Braggio D., Lee Y.-J. (2015). Targeting the notch pathway: a potential therapeutic approach for desmoid tumors. *Cancer*.

